# Publisher Correction: Paternal restraint stress affects offspring metabolism via ATF-2 dependent mechanisms in *Drosophila melanogaster* germ cells

**DOI:** 10.1038/s42003-020-0992-3

**Published:** 2020-05-18

**Authors:** Ki-Hyeon Seong, Nhung Hong Ly, Yuki Katou, Naoko Yokota, Ryuichiro Nakato, Shinnosuke Murakami, Akiyoshi Hirayama, Shinji Fukuda, Siu Kang, Tomoyoshi Soga, Katsuhiko Shirahige, Shunsuke Ishii

**Affiliations:** 1 RIKEN Cluster for Pioneering Research, Tsukuba, Ibaraki Japan; 2 PRESTO, Japan Science and Technology Agency, Kawaguchi, Japan; 3 Laboratory of Genome Structure and Function, Institute for Quantitative Biosciences, University of Tokyo, Tokyo, Japan; 4 Laboratory of Computational Genomics, Institute for Quantitative Biosciences, University of Tokyo, Tokyo, Japan; 5 Institute for Advanced Biosciences, Keio University, Tsuruoka, Yamagata Japan; 6 Systems Biology Program, Graduate School of Media and Governance, Keio University, Fujisawa, Kanagawa Japan; 7 Transborder Medical Research Center, University of Tsukuba, Tsukuba, Ibaraki Japan; 8 Department of Bio-Systems Engineering, Graduate School of Science and Engineering, Yamagata University, Yonezawa, Yamagata Japan

**Keywords:** Epigenetics, Metabolism, Heritable quantitative trait, Neuroscience

Correction to: *Communications Biology* 10.1038/s42003-020-0935-z, published online 4 May 2020.

In the original published version of the article, co-author Katuhiko Shirahige was incorrectly indicated as a jointly supervising author instead of Ki-Hyeon Seong. The error has been corrected in the HTML and PDF versions of the article.

