# Supplementary material for: Paternal restraint stress affects offspring metabolism via ATF-2 dependent mechanisms in Drosophila melanogaster germ cells
Source: Commun Biol. 2020 May 4;3:208. doi: 10.1038/s42003-020-0935-z (PMC7198565; doi:10.1038/s42003-020-0935-z)
Supplement: Supplementary file 1 — Supplementary Information [file 42003_2020_935_MOESM1_ESM.pdf]

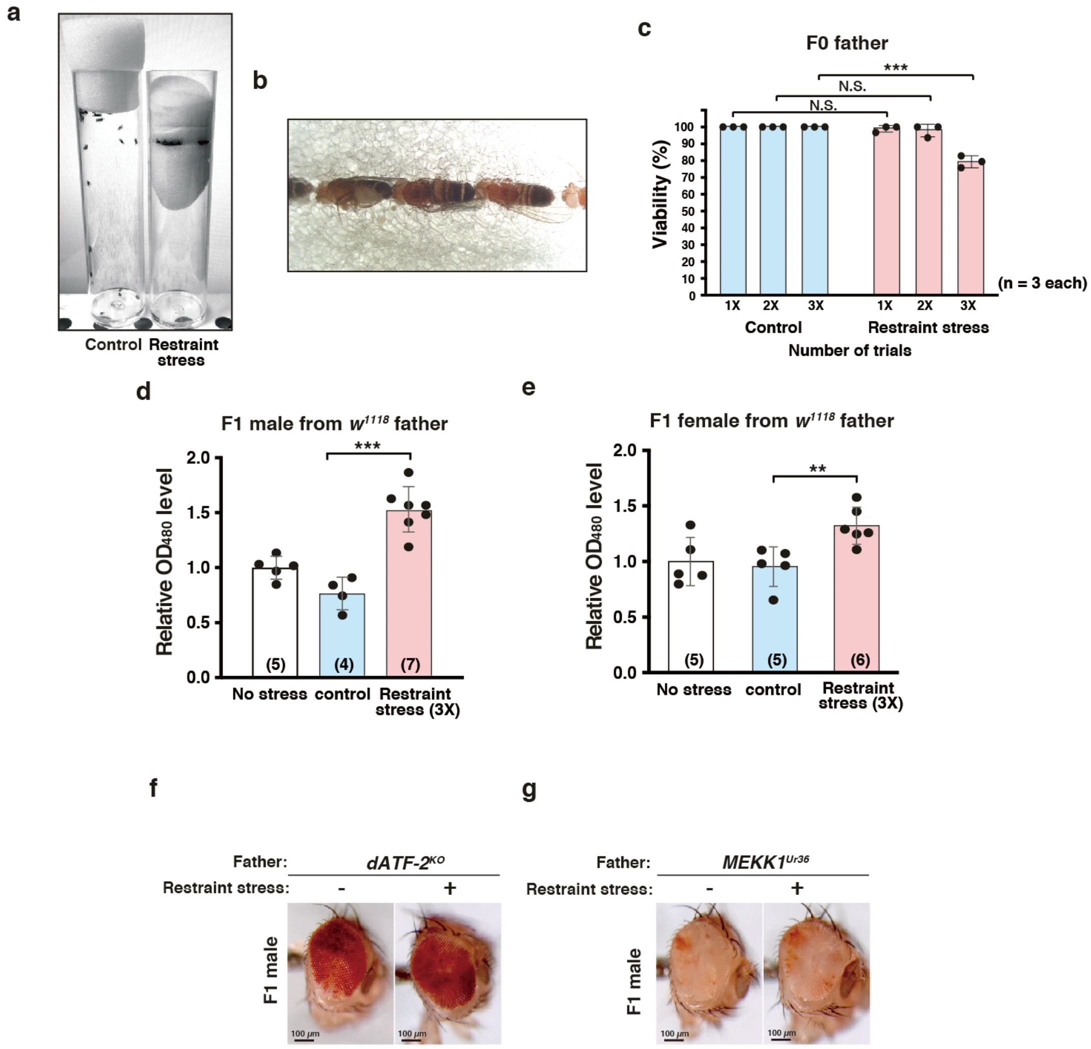

Supplementary Fig. 1 Paternal restraint stress reduces w gene expression in both sexes of w^{m4} offspring. a To induce restraint stress, a soft sponge plug was inserted into a plastic vial and CO_2-anesthetized males (20 flies) were placed on the surface of the sponge and fixed very gently using another soft sponge plug inserted from the top of the vial (right). Flies were also placed into vials not containing fly media as controls (left). **b** An enlarged photo of a fly 'sandwich'. **c** Viability of flies at 12 h after restraint stress treatment. Averages with standard deviation (s.d.) are shown (n = 3; ***p < 0.001; N.S., no significant difference, Student's unpaired t-test). **d–e** Red-eye pigment levels were measured in F1 male (**d**) and female (**e**) progeny derived from three paternal conditions (no stress, control, and restraint stress). (**d, e**) The values of red-eye pigment represent relative to each control. Averages with s.d. are shown (***p < 0.001; **p < 0.01; *p < 0.05; N.S., no significant difference, Student's unpaired t-test). Number of flies analyzed indicated in parentheses on each graph. **f, g** Eye phenotypes of F1 w^{m4} males derived from $dATF\text{-}2^{KO}$ (**f**) and $Mekk^{1Ur36}$ (**g**) father with/without restraint stress. Note that F1 offsprings from $dATF\text{-}2^{KO}$ mutant father exhibit high eye-pigment level as like same as $dATF\text{-}2^{PB}$ previously reported[1], and the eye-pigment level did not increase further in offsprings from restraint stress-exposed fathers.

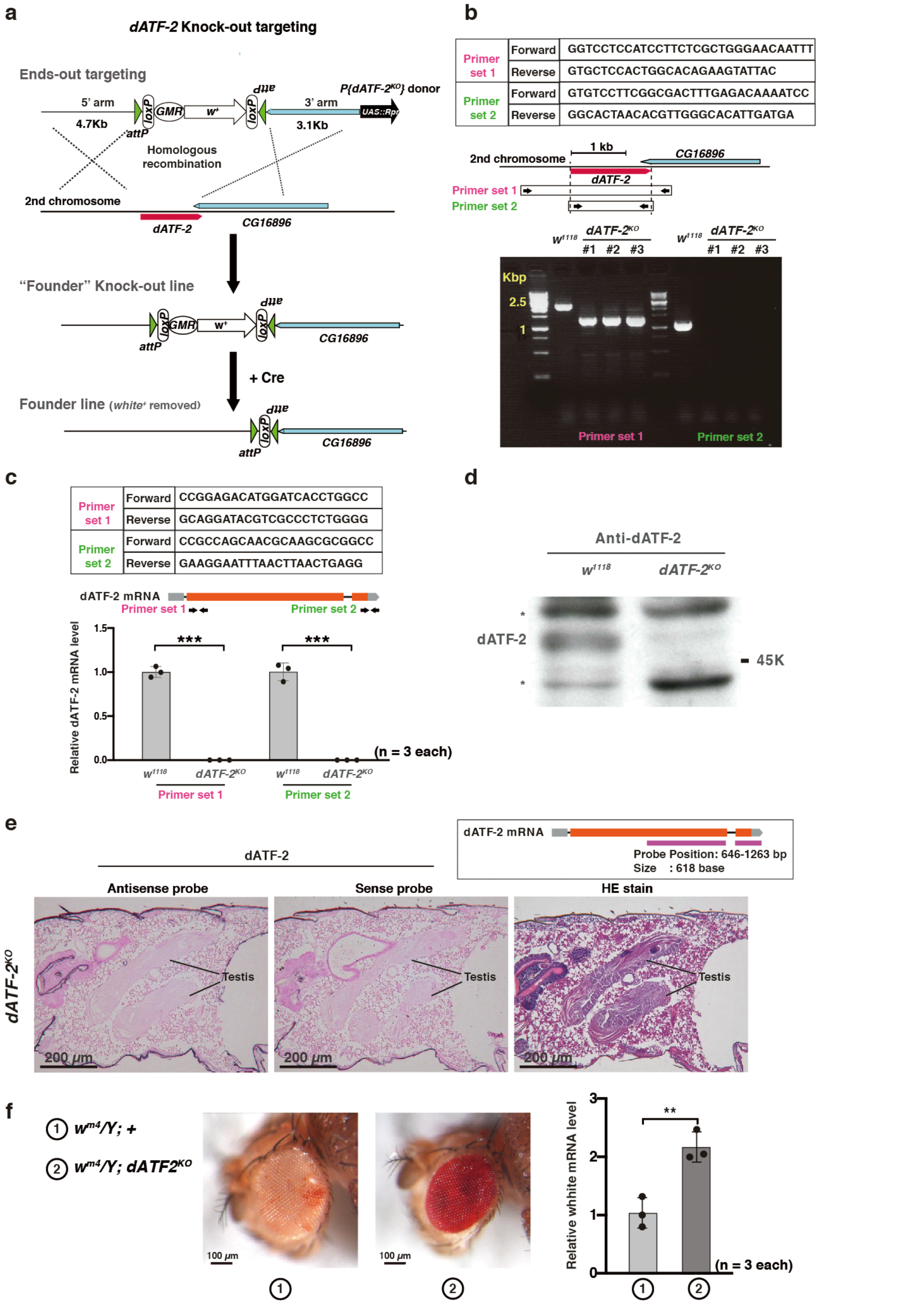

Supplementary Fig. 2 Generation and validation of *dATF-2* knockout flies. a Schematic outline of ends-out targeting of the *dATF-2* gene locus. **b** Confirmation of the complete loss of the *dATF-2* gene locus from the genome of the newly generated *dATF-2*^{KO} line using two sets of PCR primers. **c** qRT-PCR analysis of *dATF-2* mRNA using two primer sets. The data represent the mean ± s.d. (n = 3 each) with *P*-values from Student's unpaired *t*-test: ***$p <$ 0.001. **d** Western blot analysis of the *dATF-2*^{KO} strain using anti-dATF-2 antibody. Asterisks indicate non-specific bands. **e** *In situ* RNA hybridization of *dATF-2* mRNA from the *dATF-2*^{KO} strain. **f** Eye phenotypes and *white* gene expression levels of w^{m4} flies in wild-type and *dATF-2*^{KO} mutant background. The data represent the mean ± s.d. (n = 3 each) with *P*-values from Student's unpaired *t*-test: **$p <$ 0.01.

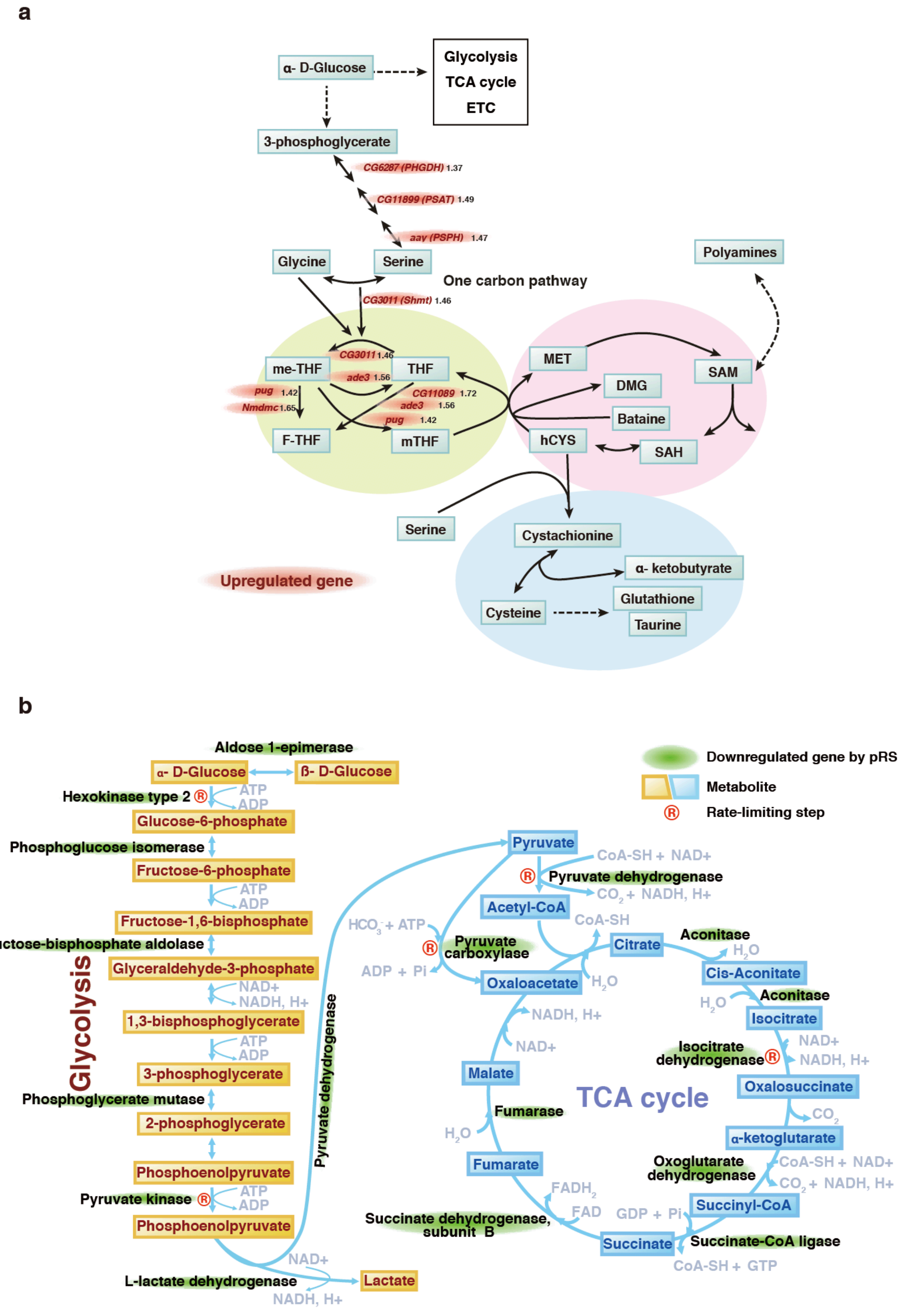

Supplementary Fig. 3 Schematic maps of metabolic pathways. a Schematic map of the one-carbon metabolic pathway and related pathways. Genes upregulated by paternal restraint stress are marked by a red ellipse. Numbers on the right of the gene name indicate the fold change in gene expression caused by paternal restraint stress. **b** Overview of glycolysis and TCA cycle processes. Genes downregulated by paternal restraint stress are marked by a green ellipse, and the red circled R indicates the rate-limiting step of each process.

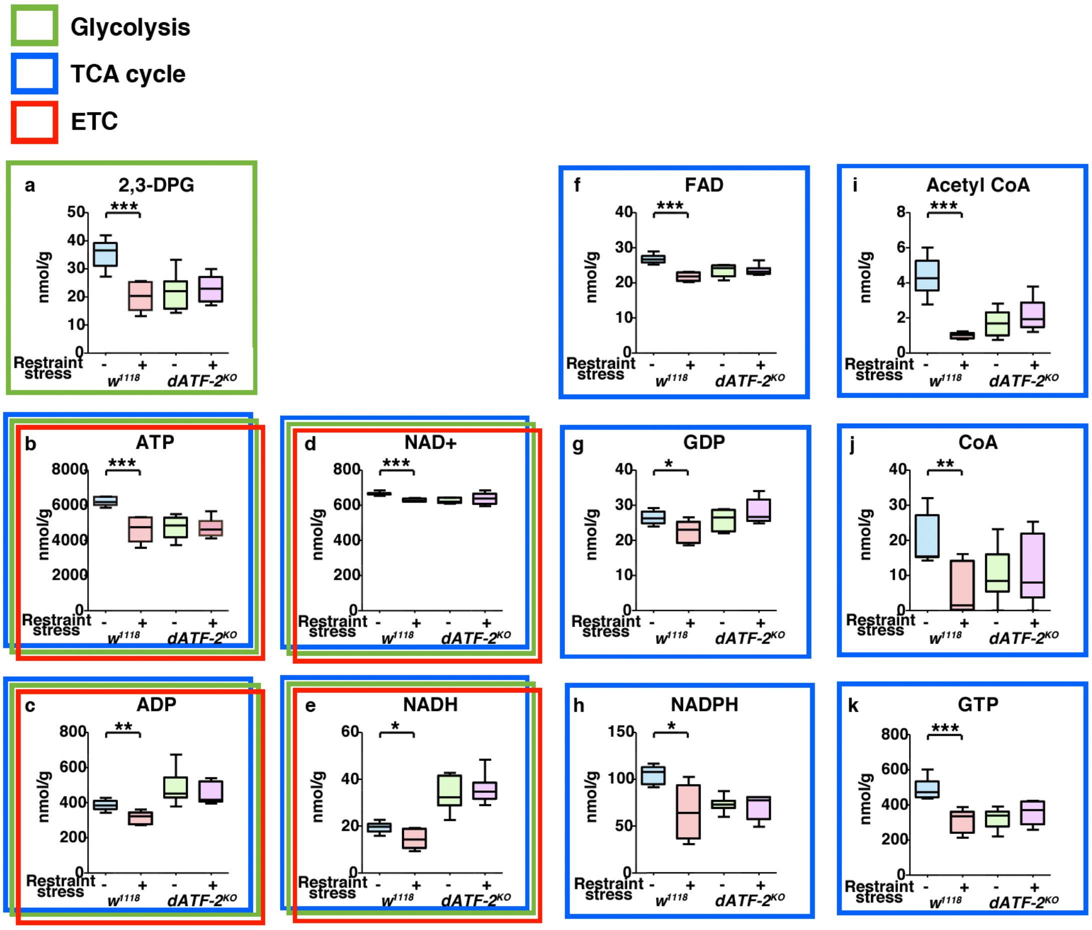

Supplementary Fig. 4 Quantitative results of the identified metabolites from the metabolome analysis. a–k Levels of metabolites involved in glycolysis (enclosed by a green square), the TCA cycle (enclosed by a blue square), and the electron transport chain (ETC; enclosed by a red square) significantly decreased in F1 flies following paternal restraint stress treatment of w^{1118} but not $dATF\text{-}2^{KO}$ fathers. Diagrams show the results of 2.3-DPG **(a)**, ATP **(b)**, ADP **(c)**, NAD+ **(d)**, NADH **(e)**, FAD **(f)**, GDP **(g)**, NADPH **(h)**, acetyl CoA **(i)**, CoA **(j)**, and GTP **(k)**, respectively. Box and whisker plots show the minimum, median, and maximum value (n = 6). Statistical significance is indicated by ***p < 0.001; **p < 0.01; *p < 0.05 (Student's unpaired t-test).

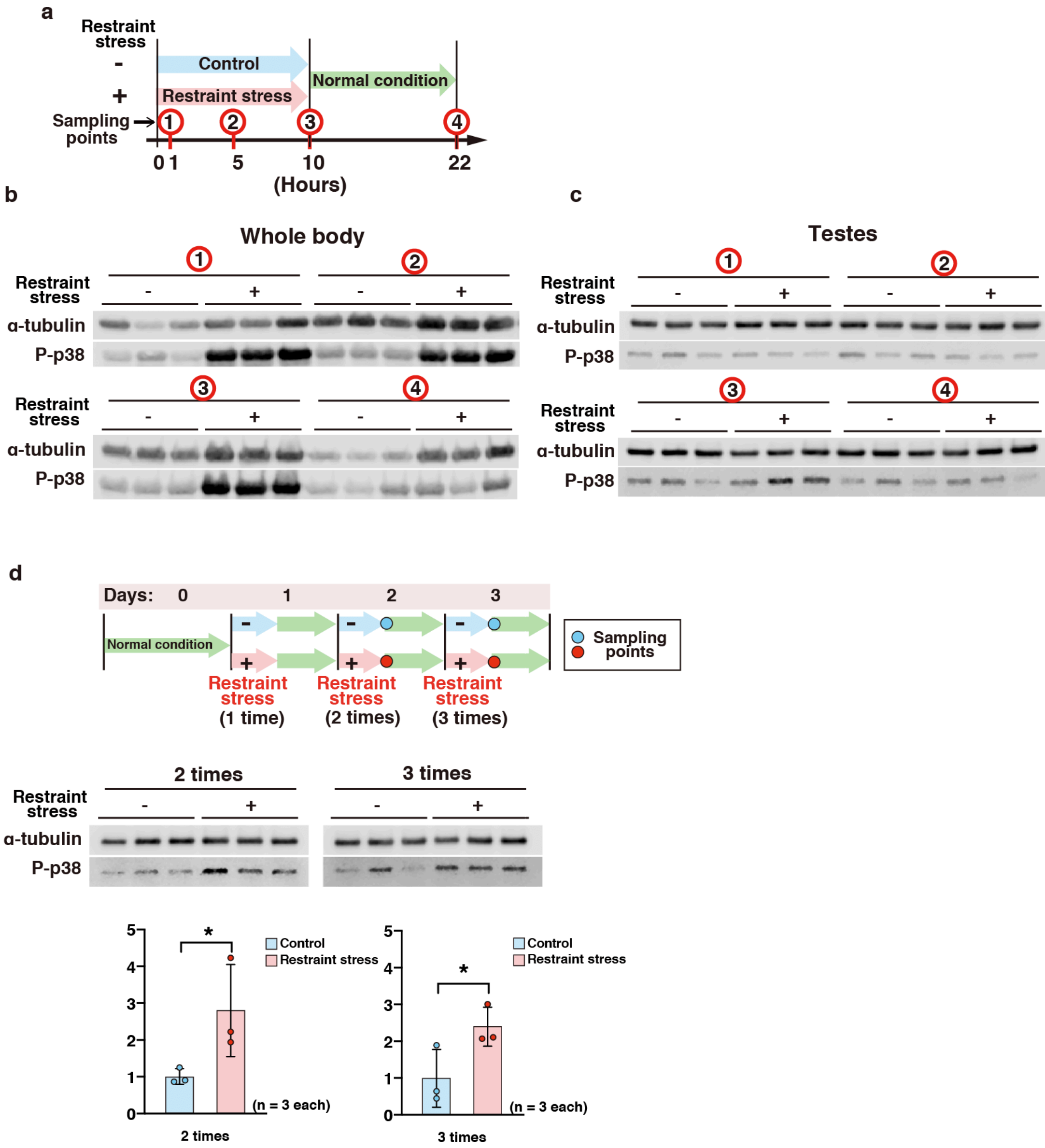

Supplementary Fig. 5 Detection of p38 phosphorylation induced by restraint stress. **a** The schematic diagram shows the developmental stages of spermatogenesis (lower diagram). **b, c** Western blot data of P-p38 MAPK and α-tubulin in whole body **(b)** and testes **(c)** with/without restraint stress for 1, 5, and 10 h and at 12 h after the 10 h. The quantitative results were shown in Fig. 5d, e. **d** Western blot analysis of P-p38 MAPK and α-tubulin in testes samples prepared after second and third times of restraint stress exposure. Lower panels shows a result of quantitative analysis. The data represent the mean ± s.d. (n = 3 each) with *P*-values from Student's unpaired *t*-test: *$p < 0.05$.

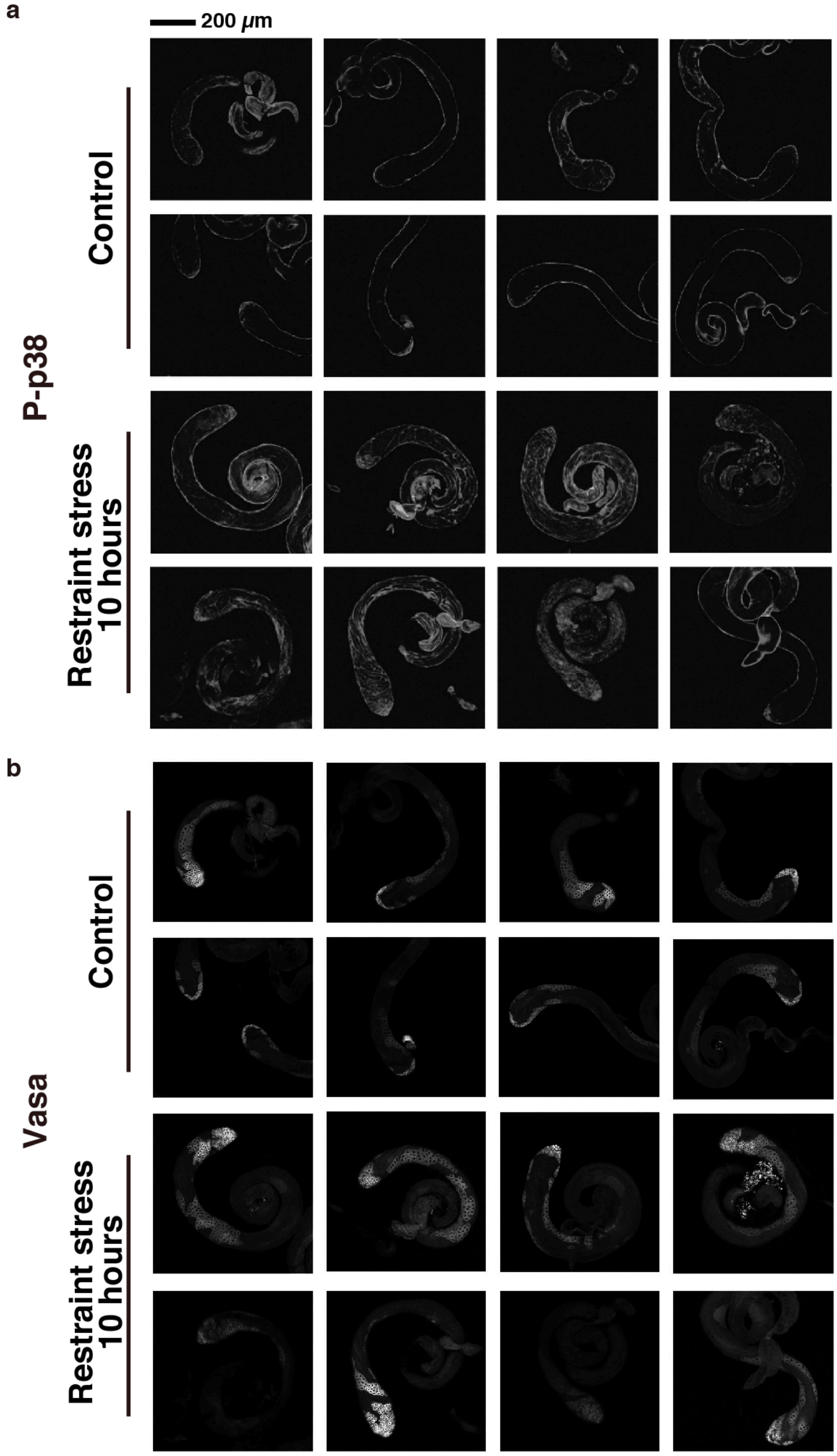

Supplementary Fig. 6 Immunohistochemical (IHC) staining of P-p38 in individual testes samples from control and restraint stress-exposed males. Immunostaining of whole mount testis preparations from restraint stress-exposed and control one-day old adult *vasa-GFP* males with anti-P-p38 **(a)** and anti-GFP **(b)**. To detect the P-p38 signal, testes were dissected from restraint stress-exposed and control males and subjected to IHC at the same time using the same reagents. After preparation of each sample, photos were taken using a confocal laser scanning microscope under the same scanning conditions.

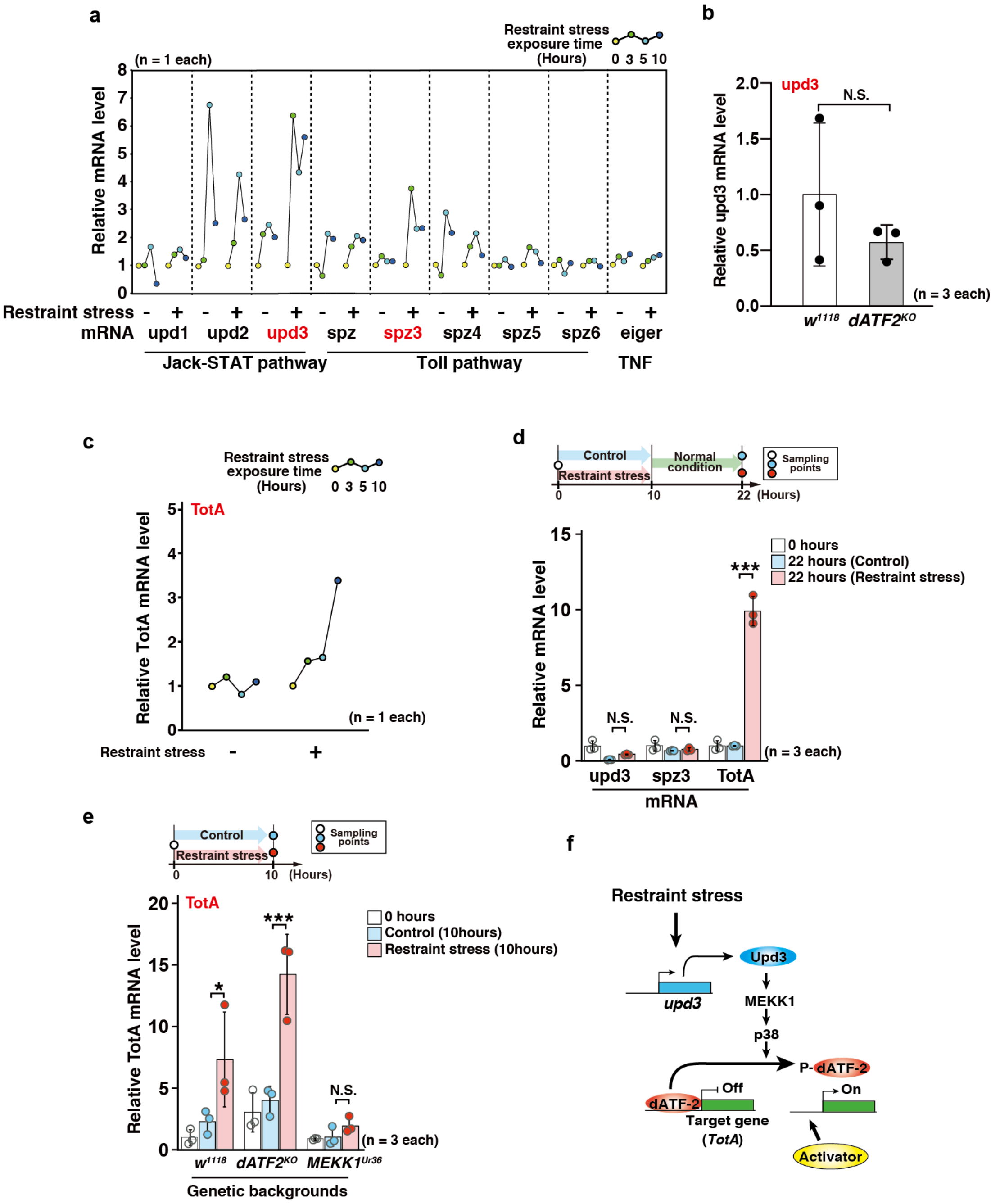

Supplementary Fig. 7 Restraint stress induces TotA expression via the MEKK1-dATF-2 pathway. a Results of screening to identify cytokines induced by restraint stress. Levels of mRNA for several cytokines were measured after 0, 3, 5, and 10 h of restraint stress exposure. Note that the results were obtained using one biological sample. **b** *upd3* gene expression levels of w^{1118} wild-type and $dATF\text{-}2^{KO}$ mutant. The data represent the mean ± s.d. (n = 3 each; N.S., no significant difference, Student's unpaired *t*-test) **c** Levels of TotA mRNA during restraint stress exposure. **d** Levels of upd3, spz3, and TotA mRNA after 12 h of recovery from the 10 h restraint stress treatment. The data represent the mean ± s.d. (n = 3 each; ***$p < 0.001$; N.S., no significant difference, Student's unpaired *t*-test). **e** Relative TotA mRNA levels during restraint stress exposure in $dATF\text{-}2^{KO}$ and $Mekk^{1Ur36}$ mutant strains. The data represent the mean ± s.d. (n = 3; ***$p < 0.001$; *$p < 0.05$; N.S., no significant difference, Student's unpaired *t*-test). **f** Schematic diagram of the model.

Supplementary Table 1. Summary of RNA-seq analysis of head samples of F1 flies

Up-regulated genes

Paternal genotype	*W*^{1118}	*dATF-2*^{KO}
pRS > C (1.4 fold-up, FDR <0.05)	7	2
Number of common gene	0	

Down-regulated genes

Paternal genotype	*W*^{1118}	*dATF-2*^{KO}
pRS < C (1.4 fold-down, FDR <0.05)	2	6
Number of common gene	0	

Supplementary Table 2. Primer sets used for qRT-PCR

Gene name	Forward primer (5'-3')	Reverse primer (5'-3')
rp49	GATGACCATCCGCCCAGCATAC	AGTAAACGCGGTTCTGCATGAGC
upd1	TCTGGTTGCAGTTGCATTCG	GGGTTACTGGGTGGGATTGG
upd2	CCCTGGAGTACGGCAATCTG	CATGTGGCGGTACCAAGTCT
upd3	CTACAGATTCCTGCCCCGTC	GTTGCGCATGTACGTGAAGG
TotA	CCCTGAGGAACGGGAGAGTA	CTTTCCAACGATCCTCGCCT
spz	ACGTACGAGGCCAAGGAGTA	GGGTATGGGCATGAAGGGTG
spz3	GGTGCCGCTCTTCTCCTATC	CACATCCAGTCCCAAAGCCA
spz4	ACTGCAGACGATTTGCGATTG	CAGATTTCACACTCGCACGC
spz5	AGGAAAGACGTACTGCGAGC	TTCGTCCACCAACAGAGTGG
spz6	AAGAAGCAAACGAGCGGAGA	CCTCACCGTAGTCAGATGGC
eiger	ACTTCCAAAGAGAGCCCTGC	AGTCCTCGGATCTGGCTGAA
dATF-2-1	CCGGAGACATGGATCACCTGGCC	GCAGGATACGTCGCCCTCTGGGG
dATF-2-2	CCGCCAGCAACGCAAGCGCGGCC	GAAGGAATTTAACTTAACTGAGG

Sapplementary Refference

1. Seong, K. H., Li, D., Shimizu, H., Nakamura, R. & Ishii, S. Inheritance of stress-induced, ATF-2-dependent epigenetic change. *Cell* **145**, 1049-1061, doi:10.1016/j.cell.2011.05.029 (2011).
