# Supplementary material for: Paternal restraint stress affects offspring metabolism via ATF-2 dependent mechanisms in Drosophila melanogaster germ cells
Source: Commun Biol. 2020 May 4;3:208. doi: 10.1038/s42003-020-0935-z (PMC7198565; doi:10.1038/s42003-020-0935-z)
Supplement: Supplementary file 2 — Description of Additional Supplementary Items [file 42003_2020_935_MOESM2_ESM.pdf]

Supplementary Data 1. Source data for figures.

Excel file containing source data for all main and supplementary figures.
