# Supplementary material for: Paternal restraint stress affects offspring metabolism via ATF-2 dependent mechanisms in Drosophila melanogaster germ cells
Source: Commun Biol. 2020 May 4;3:208. doi: 10.1038/s42003-020-0935-z (PMC7198565; doi:10.1038/s42003-020-0935-z)
Supplement: Supplementary file 4 — Reporting Summary [file 42003_2020_935_MOESM4_ESM.pdf]

natureresearch

Corresponding author(s): Ki-Hyeon Seong or Shunsuke Ishii

Last updated by author(s): Mar 28, 2020

Reporting Summary

Nature Research wishes to improve the reproducibility of the work that we publish. This form provides structure for consistency and transparency in reporting. For further information on Nature Research policies, see Authors & Referees and the Editorial Policy Checklist.

Statistics

For all statistical analyses, confirm that the following items are present in the figure legend, table legend, main text, or Methods section.

n/a	Confirmed	
☐	☒	The exact sample size (*n*) for each experimental group/condition, given as a discrete number and unit of measurement
☐	☒	A statement on whether measurements were taken from distinct samples or whether the same sample was measured repeatedly
☐	☒	The statistical test(s) used AND whether they are one- or two-sided<br>*Only common tests should be described solely by name; describe more complex techniques in the Methods section.*
☒	☐	A description of all covariates tested
☒	☐	A description of any assumptions or corrections, such as tests of normality and adjustment for multiple comparisons
☒	☐	A full description of the statistical parameters including central tendency (e.g. means) or other basic estimates (e.g. regression coefficient) AND variation (e.g. standard deviation) or associated estimates of uncertainty (e.g. confidence intervals)
☒	☐	For null hypothesis testing, the test statistic (e.g. *F*, *t*, *r*) with confidence intervals, effect sizes, degrees of freedom and *P* value noted<br>*Give P values as exact values whenever suitable.*
☒	☐	For Bayesian analysis, information on the choice of priors and Markov chain Monte Carlo settings
☒	☐	For hierarchical and complex designs, identification of the appropriate level for tests and full reporting of outcomes
☒	☐	Estimates of effect sizes (e.g. Cohen's *d*, Pearson's *r*), indicating how they were calculated

Our web collection on statistics for biologists contains articles on many of the points above.

Software and code

Policy information about availability of computer code

Data collection	Western blotting imaging was acquired with the software for the Image studio Version 4.0.21.<br>Confocal fluorescent imaging was acquired with Zen Software (Zeiss).<br>We used our newly developed software to automatically determine the timing of death in rotenone stress test (Seong et al., manuscript in preparation). The software code will be available after the manuscript of the method is published.
Data analysis	All statistical analyses have used Prism 8 software.<br>Metabolome analysis were conducted using a web-based application (Xia et. al., 2010 and 2011).

For manuscripts utilizing custom algorithms or software that are central to the research but not yet described in published literature, software must be made available to editors/reviewers. We strongly encourage code deposition in a community repository (e.g. GitHub). See the Nature Research guidelines for submitting code & software for further information.

Data

Policy information about availability of data

All manuscripts must include a data availability statement. This statement should provide the following information, where applicable:

- Accession codes, unique identifiers, or web links for publicly available datasets
- A list of figures that have associated raw data
- A description of any restrictions on data availability

RNA-seq data are accessible in the NCBI Gene Expression Omnibus (GEO; https://www.ncbi.nlm.nih.gov/geo/) under the accession number GSE140950.
All datasets generated and analyzed during the current study are available from the corresponding author on reasonable request.

Field-specific reporting

Please select the one below that is the best fit for your research. If you are not sure, read the appropriate sections before making your selection.

☒ Life sciences ☐ Behavioural & social sciences ☐ Ecological, evolutionary & environmental sciences

For a reference copy of the document with all sections, see nature.com/documents/nr-reporting-summary-flat.pdf

Life sciences study design

All studies must disclose on these points even when the disclosure is negative.

Sample size	No sample size calculation was performed for each experiment. The sample size for each experiment was determined based on previous literatures.
Data exclusions	No data were excluded.
Replication	Replicate experiments were successful.
Randomization	n/a
Blinding	n/a

Reporting for specific materials, systems and methods

We require information from authors about some types of materials, experimental systems and methods used in many studies. Here, indicate whether each material, system or method listed is relevant to your study. If you are not sure if a list item applies to your research, read the appropriate section before selecting a response.

Materials & experimental systems

n/a	Involved in the study	
☐	☒	Antibodies
☒	☐	Eukaryotic cell lines
☒	☐	Palaeontology
☐	☒	Animals and other organisms
☒	☐	Human research participants
☒	☐	Clinical data

Methods

n/a	Involved in the study	
☒	☐	ChIP-seq
☒	☐	Flow cytometry
☒	☐	MRI-based neuroimaging

Antibodies

Antibodies used	Anti-Phospho-p38 antibody, for Cell Scignaling Technology, Cat. No. 9215 Lot. 7<br>Anti-dATF-2 antibody (created in our laboratory)<br>Anti-alpha-tubulin antibody, sigma, Cat. No. T6199<br>12A6 anti-GFP antibody, DSHB, Cat. No.DSHB-GFP-12A6<br>Anti-histone H3 antibody, Abcam, Cat. No.ab1791, Lot. GR89010-1<br>Anti-histone H3K9me2, Abcam, Cat. No.ab1220, Lot. GR32351-2
Validation	The antibodies used in this study are used in previous studies, and the blotting and staining pattern has been analyzed well. Anti-dATF-2 antibody, created in our laboratory, also repeatedly confirmed specific recognition of dATF-2 itself (Sano et. al., 2005; Okamura et al.,2007; Shimizu et al., 2008, Seong et al., 2011).

Animals and other organisms

Policy information about studies involving animals; ARRIVE guidelines recommended for reporting animal research

Laboratory animals	The strains used in this study were w1118 (WT; BDSC 5905), In(1) wm4 (wm4 for short)(25), Mekk1Ur36(26), elav-GS-GAL4 (BDSC 43642) (27), and UAS-upd3 (Harvard EXELIXIS stock collection P[XP]d04951) (28). To generate the dATF-2KO null mutant, the ends-out targeting approach was used (29-31). The dATF-2KO null mutant lacks the entire dATF-2 gene region and does not express dATF-2 (Supplementary Figure S2). To normalize the genetic background, wm4 and dATF-2KO were backcrossed six times with w1118.
Wild animals	n/a

Field-collected samples	n/a
Ethics oversight	n/a

Note that full information on the approval of the study protocol must also be provided in the manuscript.
